# Parent and Physician Preference for Anxiolytic Medication Prior to Laceration Repair in Young Children

**DOI:** 10.7759/cureus.32412

**Published:** 2022-12-11

**Authors:** Muhammad Waseem, Hina Asad, Masood A Shariff, Eric Epstein, Yusif Umar, Mark Leber

**Affiliations:** 1 Emergency Medicine, New York City (NYC) Health and Hospitals Lincoln Medical Center, New York, USA; 2 Anesthesia, St. Barnabas Hospital Health System, New York, USA; 3 Emergency Medicine, The Brooklyn Hospital Center, New York, USA

**Keywords:** pain management, parent-provider communication, emergency room pediatric, anxiolytic, pediatric laceration repair

## Abstract

Objectives

Pediatric laceration repair is a daunting process for parents and physicians. The repair could take place quickly if the child is calm and relaxed.This study aimeds to evaluate parental and physician preference for anxiolytic medication administration prior to laceration repair, with a pre-and post-repair survey on parents’ and physicians’ initial preference and follow-up perception.

Methods

Parents or guardians of children aged six months to five years who presented with simple lacerations and their physicians were asked to complete a survey on potential benefits and expectations of anxiolytic use before and after the laceration repair.

Results

Fifty parents/guardians completed the survey. Forty-three (86%) expressed their preference for anxiolytic medication use if it had been available, before laceration repair. Parents/guardians perceived reactions to laceration repair before and after the procedure were significant, ranging from “uncontrolled crying” to “continuous crying” (p=.032). The parents/guardians overwhelmingly preferred to take part in the decision-making process during the repair (not significant). Preference for anxiolytic use was high before repair at 54% and increased to 62% after witnessing the procedure (not significant). Physicians who completed the survey supported the use of anxiolytics 84% of the time. Forty (80%) physicians preferred the intranasal route, while parents/guardians preferred the oral route (58%).

Conclusions

Procedural sedation is critical for anxiety control and to minimize the difficulties related to treatment. In our study, parents and physicians supported the administration of an anxiolytic agent to help alleviate anxiety and achieve optimal outcomes.

## Introduction

Procedural sedation and analgesia (PSA) are commonly performed in the emergency department (ED). However, it is often underused in cases involving children. The basic purpose of sedation in minor cases is to have a better outcome during the procedure and to improve patient comfort [1,2]. Children often receive no anxiolytic medication for minor frightening procedures when local anesthesia is administered. However, significant patient anxiety around manipulating the painful injury, using needles, etc., is not usually addressed [3]. Now, more parents and caregivers are opting for the use of sedative drugs and consider sedation as a safe intervention [4]. Indeed, children experience significant anxiety even during minor procedures. Adequate anxiety control with effective and safe procedural sedation is important for successful laceration repair.

In this study, we aimed to determine parental and physician preferences and expectations of effectiveness in the use of anxiolytic medications while performing laceration repair and whether their preferences change after observing their child undergo the laceration repair procedure.

## Materials and methods

This study is a prospective survey of parents of children ages six months to five years who presented to the ED with superficial lacerations of size measuring up to 5 cm and requiring single-layer closure. Children presenting with a head injury and loss of consciousness, severe trauma with suspected internal injuries, wounds requiring more than a layered closure (scar revision/debridement/extensive undermining/use of stents or retention sutures), as well as lacerations with an underlying fracture or tendon injury, and additionally, administration of any anxiolytic (i.e., midazolam) during any point during their current ED visit, were excluded from this study. As part of the standard of care, pediatric patients received local anesthesia (1% lidocaine with or without 1:100,000 epinephrine) in the repair of the laceration. While awaiting the laceration repair procedure on their child, all parents received an information sheet with risks, benefits, and options for anxiolytics for uncomplicated laceration repairs. It also included information about other pharmacologic options, for example, the administration of intranasal midazolam [5]. At no point during the pediatric patient’s care had they received any anxiolytic treatment. This study protocol was approved by the Institutional Review Board committee (approval no. 13-012).

The parent/guardian and physicians who consented to participate in this study received a two-part survey to complete anonymously, prior to and following the laceration repair. The research team consisted of ED physicians, ED pediatric physicians, and residents. They reviewed the protocol and used an IRB-approved script to present the study to the parents/guardians of children who presented with lacerations. This includes the risks and benefits of anxiolytic use. The physicians who performed the laceration repair were either senior residents (pediatric, emergency medicine, or oral surgery) or pediatric attending physicians who had experience in laceration repair. The child was restrained by both parental and support staff to allow the repair to proceed. These physicians completed a pre-and post-repair survey questionnaire as well.

The parent/guardian survey (see Appendix) included the child’s age and gender, parent/guardian’s demographic information, prior history of laceration repair, expected child’s behavior during repair, preference of anxiolytic use, anticipated outcome and rationale, preferences of both the route of administration and their involvement in the child’s medical decision-making process. Post-laceration repair questions referred to the child’s behavior during the repair procedure, preference for anxiolytic use, and preference for being involved in the medical decision-making process. The service-rendering physicians’ survey consisted of questions regarding the preferred route of administration of anxiolytic and the child’s expected behavior with and without anxiolytic administration pre- and post-repair. The standard of care procedure for laceration repair was irrigation, local anesthesia, and primary closure with sutures or staples. No anxiolytic medication was provided to any of the study participants before or following laceration repair.

Statistical analysis

Data were analyzed using Stata SE 14 software (Stata Corp. LLC, College Station, Texas, USA). Categorical data related to demographic variables are presented as frequencies and proportions. Associations between the independent variables and the primary outcomes (parental and physician preferences in the use of anxiolytic medications before performing simple laceration repair) were tested using a t-test or chi-square test as appropriate. The level of significance was set to 0.05 (two-tailed).

## Results

A total of 120 parents/guardians were approached for the study. Fifty parents/guardians met the inclusion criteria and participated in the survey. Seventy parents/guardians met the following exclusion criteria: 25 had children who were over the age of five; 11 parents declined to participate; 14 did not complete the second part of the survey or were unable to complete the survey; eight had children with deep laceration wound; seven were transferred to another facility; five decided later not to participate. Demographic information is presented in Table 1. Children had a median age of three years with a range of one to five years, and the majority were female (52%). The children who presented to the ED were with their mother (80%) or father (18%) and included the following ethnic backgrounds: Latino (56%), and Black (44%). The parents were of the following age range: 22 to 25 years (30%) followed by 26 to 30 years (28%), and 31 to 40 years (18%). Their education levels were as follows: 42% had a high-school education, 28% had not completed high school, and 10% were college graduates. The majority were employed full-time (40%) and 24% held part-time jobs. Wound location ranged from the face (74%), lip (14%), and scalp (8%) to the leg (2%). Additionally, 12% of the children had a history of previous lacerations that were repaired with a local anesthetic, and two of those patients had experienced a reaction to medications. The child’s reaction prior to laceration repair was noted with occasional crying and parents stated that no medication was given prior to repair.

**Table 1:** Characteristics of children and parents Data are presented as frequency (percent) for categorical and mean ±standard deviation for continuous. SD: Standard deviation

Characteristics	N (%)
Children	
Male	24 (48.0%)
Female	26 (52.0%)
Age in years, mean+SD	3.1±1.2
Median (Range)	3 (1-5)
Parent/Guardian	
Male	11 (22.0%)
Female	39 (78.0%)
Age range	
18-21	6 (12.0%)
22-25	15 (30.0%)
26-30	14 (28.0%)
31-40	9 (18.0%)
41-50	4 (8.0%)
51-older	1 (2.0%)
Relationship to Child	
Mother	40 (80.0%)
Father	9 (18.0%)
Grandfather	1 (2.0%)
Primary Language	
English	24 (48.0%)
Spanish	25 (50.0%)
Other	1 (2.0%)
Ethnicity	
Black or African American	22 (44.0%)
Hispanic or Latino	28 (56.0%)
Employment Status	
Full time	20 (40.0%)
Part-time	12 (24.0%)
Self-employed	2 (4.0%)
Unemployed/looking for work	6 (12.0%)
Housewife/husband	9 (18.0%)
Student	1 (2.0%)
Education Level of Parent/Guardian	
Less than high school	14 (28.0%)
High School	21 (42.0%)
Vocational / Technical school (2 years)	1 (2.0%)
Some College	4 (8.0%)
2 Year College	4 (8.0%)
4 Year College	5 (10.0%)
Masters	1 (2.0%)
Current Presentations Wound Location	
Face	37 (74.0%)
Lip	7 (14.0%)
Scalp	4 (8.0%)
Legs	1 (2.0%)
Prior Allergic Reaction	2 (3.9%)
History of Previous Wounds Requiring Repair	5 (10.0%)
Previous Wound Repair Technique	
Stitches/Suture	4 (8.0%)
Glue	1 (2.0%)
Staples	0

Parents stated an overall acceptance of the administration of anxiolytic agents before laceration repair (86%), if offered by the primary team, and preferred the oral route of administration (Table 2). Overall, the preference for the use of anxiolytics increased by 19% after witnessing the laceration repair. After the repair in the ED, the parent/guardians were asked to assume a situation where “anxiolytic was given” and the laceration repair question was repeated. Results showed a shift from “Uncontrollable crying and fighting” of 29% to 14% with “Continuous crying and/or fighting” from 39% to 51%; some parents/guardians changed their response of “Occasional crying” from 14% to 29% (Table 3). Among the parents who declined the anxiolytic use, their reasons ranged from the risk of an allergic reaction, vomiting, and respiratory distress. The parent/guardian's decision on whether or not to administer an anxiolytic agent did not change significantly following observation of the laceration repair procedure.

**Table 2:** Parent/guardian responses prior to laceration repair IM: Intramuscular, IV: Intravenous

Responses	N (%)
Parents prefer to use anxiolytics during wound repair	
Yes	43 (86%)
No	7 (14%)
Reason for refusal	
It will not work	4 (57.1%)
It will be more expensive	0
Risk of an allergic reaction	5 (71.4%)
Risk of vomiting	1 (14.3%)
Risk of problems breathing	1 (14.3%)
Must stay longer in the hospital	0
Other	4 (57.1%)
Parents prefer to be inside the room during the procedure	
Yes	45 (90.0%)
No	5 (10.0%)
Expectation if anxiolytics is used during the repair	
Cooperative or sleeping	26 (52.0%)
Occasional crying	16 (32.0%)
Continuous crying and/or fighting	3 (6.0%)
Uncontrollable crying and fighting	5 (10.0%)
Parent's preference on the route of administration of anxiolytics	
Oral	29 (58.0%)
Intranasal	14 (28.0%)
IM	3 (6.0%)
IV	1 (2.0%)
None	3 (6.0%)

**Table 3:** Survey of parents/guardians' responses before and after laceration repair

Parent responses before and after wound repair	Before Repair	After Repair	P-value
Preference for the use of anxiolytics			0.817
I would definitely want the medicine	27 (54.0%)	31 (62.0%)	
I might want the medicine	19 (38.0%)	15 (30.0%)	
I do not care if we use the medicine	0	0	
I do not think I want the medicine	3 (6.0%)	2 (4.0%)	
I definitely do not want the medicine	1 (2.0%)	1 (2.0%)	
Parents perceived reaction to the child before and after laceration repair			0.032
Cooperative or sleeping	7 (14.0%)	2 (4.0%)	
Occasional crying	7 (14.0%)	14 (28.0%)	
Continuous crying and/or fighting	20 (40.0%)	26 (52.0%)	
Uncontrollable crying and fighting	15 (30.0%)	7 (14.0%)	
How would you prefer decision-making capacity to be carried out?			0.791
I think the emergency room doctor should definitely make the decision if my child should get calming medicine through the nose	17 (34.0%)	17 (34.0%)	
I would like to help the emergency room doctor make the decision if my child should get a calming medicine through the nose	24 (47.1%)	18 (35.3%)	
I alone should make the decision whether or not my child should get a calming medicine through the nose	9 (17.6%)	8 (15.7%)	
How strongly do you feel about getting involved in making medical decisions for your child?			0.53
I strongly prefer not to get involved	2 (4.0%)	5 (10.0%)	
I prefer not to get involved	10 (20.0%)	8 (16.0%)	
Neutral	1 (2.0%)	1 (2.0%)	
I prefer to get involved	17 (34.0%)	18 (36.0%)	
I strongly prefer to get involved	20 (40.0%)	12 (24.0%)	

Out of the 50 respondents that completed the pre- and post-procedure portions of the survey, 46 parents/guardians (92%) who had selected the use of anxiolytics pre-procedure maintained their favorable decision to use anxiolytics post-procedure as well per the responses to the questions before and after observing the child undergo laceration repair. A McNemar-Bowker test was conducted on the results, indicating no statistical significance (p= 0.625) to parents changing their minds pre- and post-procedure.

The results from the survey completed by the treating physicians are presented in Table 4. The physicians were equally willing to help parents in their decision-making capacity and to take sole responsibility for the patient’s care. Physicians answered their expectation of anxiolytics during wound repair as having a child that would react with “Occasional crying” (56%), and without anxiolytics would involve having a child with “Continuous crying and/or fighting” (64%), which correlated with 53% after the procedure followed by 31% as “Occasional crying.” The physicians were willing to utilize the anxiolytics during the procedure, if available (93% of respondents). Forty physician responses indicated their preference for an intranasal route for anxiolytic delivery (89%), while only five (11%) preferred the oral route.

**Table 4:** Treating physician survey responses (N=45) IM: Intramuscular, IV: Intravenous

Physician Responses	N (%)
Physician’s take on decision-making capacity to be carried out	
I think the emergency room doctor should definitely make the decision if the patient should get calming medicine through the nose	24 (53.3%)
I would like help from the parent/legal guardian to make the decision if the child should get a calming medicine through the nose	20 (44.4%)
The parent/legal guardian alone should make the decision whether or not their child should get a calming medicine through the nose	1 (2.2%)
Expectation if anxiolytics were used during wound repair	
Cooperative or sleeping	19 (42.2%)
Occasional crying	25 (55.6%)
Continuous crying and/or fighting	2 (4.4%)
Uncontrollable crying and fighting	0
Preference to use anxiolytics on pediatric patients during wound repair	
Yes	42 (93.3%)
No	3 (6.7%)
Expectation if anxiolytics is not used	
Cooperative or sleeping	2 (4.4%)
Occasional crying	6 (13.3%)
Continuous crying and/or fighting	29 (64.4%)
Uncontrollable crying and fighting	8 (17.8%)
Route (MD5)	
Oral	5 (11.1%)
Intranasal	40 (88.9%)
IM	0
IV	0
None	0
How would you describe your patient’s behavior during the wound repair?	
Cooperative or sleeping	1 (2.2%)
Occasional crying	14 (31.1%)
Continuous crying and/or fighting	24 (53.3%)
Uncontrollable crying and fighting	6 (13.3%)
Preference for the use of anxiolytics	
I definitely would administer the medicine	33 (73.3%)
I might want to administer the medicine	9 (20.0%)
I do not care if we administer the medicine	1 (2.2%)
I do not think I want to administer the medicine	0
I definitely do not want to administer the medicine	2 (4.4%)

Table 5 compares the responses of parents and physicians to the use of anxiolytic agents. If the anxiolytic agents were not used, both the parents and physicians predicted a child’s response to be “Continuous crying and/or fighting” (42% vs. 64%) followed by “Uncontrollable crying and fighting” (31% vs. 18%), respectively. Whereas the question of laceration repair with anxiolytic agent use was “Cooperative or sleeping” and “occasional crying” with parents choosing the former (58%) and physicians choosing the latter (56%). The actual reaction of the child described by the treating physician was “Continuous crying” (53%) followed by “Occasional crying” (31%).

**Table 5:** Comparison of parent/guardian and physician reaction to the laceration repair

Expectations of Child’s Behavior During Suturing, Assessed Before Repair (n=45)			
If Anxiolytic NOT Used n (%)	Parent’s expectation	Physician’s expectation	Child’s actual behavior without anxiolytic
Cooperative or sleeping	7 (15.6)	2 (4.4)	1 (2.2)
Occasional crying	5 (11.1)	6 (13.3)	14 (31.1)
Continuous crying and/or fighting	19 (42.2)	29 (64.4)	24 (53.3)
Uncontrollable crying and fighting	14 (31.1)	8 (17.8)	6 (13.3)
If Anxiolytic IS Used n (%)	Parent’s expectation	Physician’s expectation	
Cooperative or sleeping	26 (57.8)	18 (40.0)	
Occasional crying	14 (31.1)	25 (55.6)	
Continuous crying and/or fighting	2 (4.4)	2 (4.4)	
Uncontrollable crying and fighting	3 (6.7)	0	

## Discussion

Parents/guardians overwhelmingly supported the administration of anxiolytic agents to their children if they required laceration repair. Providers should consider giving anxiolytic medication in all cases. Inherent risks and potential contraindications should be discussed with the child’s parent/guardian. Furthermore, physicians preferred the use of anxiolytic medications delivered by the intranasal route to facilitate the administration of the medication. The survey also suggests that parents were not satisfied with the conventional approach of simply holding the child and preferred alternative efforts to reduce suffering. Adequate anxiolysis and analgesia are both important for pediatric patients to reduce potential suffering and facilitate the successful completion of the procedure.

The results of our survey indicate nearly unanimous endorsement of anxiolytic agents used by both ED physicians and the parents of children who participated in this study. Support for the use of anxiolytics continued following the repair procedure since no participants on the survey changed their responses by indicating they did not want it to be used in future procedures. Both the appropriate selection of PSA agents and the administration routes are important. We considered midazolam as an agent for calming the child during all procedures. This is true since such procedures required initial preparation (washing the wound) and the positioning and stabilization of the patient and suturing. These are accomplished best with a willing and calm child. The properties of midazolam are mild anxiolysis with mild sedation and amnesia when given at a low dose [6]. Furthermore, it does not persist for a prolonged period and has minimal lingering effects. The parents and physicians assumed the child to be “continually crying or fighting,” and following the procedure, their response had shifted to “occasional crying.” This may have possibly been due to the experience of the ED staff managing the child, reducing the anxiety through behavioral modification tactics. A comparison of midazolam has been done in ED studies with ketamine (N-methyl-D-aspartate (NMDA) receptor antagonist) [7] and dexmedetomidine (alpha-2 agonist) [8], which have shown benefits and comparable anxiolytic properties. The route of administration of PSA agents should be tailored to the individual patients and/or procedures. Intranasal or intravenous routes may allow for more rapid initiation of action and the facilitation of repeated dosing as required.

In children, inadequate management of distress due to anxiety may cause significant harm both in the short term and the long term. Suboptimal anxiety control can influence their reaction to future painful experiences. Childhood trauma may have lasting effects on the future mental and physical health of a child. Physical restraint has been commonly used in pediatrics but may be associated with psychological or physical trauma for the child and result in either suboptimal procedures or diagnostic test results [9]. Trauma may affect the behavior, development, and reactions of children. These events can often lead to emotional and psychological effects. Such early-age imprints can affect a child’s mental and physical health for years to come [10]. A child’s reaction to trauma may vary depending on their resiliency and age. Toddlers and young children will likely feel greater fear in response to trauma. Young children usually have not developed the ability to recognize where they can be safe. Hence their fear may extend past the circumstances of the traumatic event [6]. Mild sedatives may help to reduce both anxiety and psychological or physical trauma.

Anxiolytics used during procedures can improve outcomes. Most studies recognize the successful completion of a test or procedure as “effective.” Effective PSA during procedures not only provides relief from suffering but also frequently facilitates the successful and timely completion of the procedure [11]. In children, it may also ensure physical comfort and minimize psychological distress. Painful procedures are less traumatic if a child receives such medication at a low dose with adequate analgesia. Lowe et al. in their survey gauged parent satisfaction by exploring the theme of provider performance, anxiety, pain, and cosmesis [12]. The overall scoring in the study favored provider performance and their care in handling the laceration repair while the parent’s self-reported anxiety about the procedure was high; it was concluded that a team-based approach to the repair, balanced the parent’s fear and reaction. The anxiety parents experienced before and after the procedure in our study was related to the question “if anxiolytic was not used,” where parents and physicians chose “continuous crying” as a reaction to the uncertainty of the procedure by not having some calming medication onboard. Another study by Crumm et al. surveyed patients, caregivers, and physicians for pain and anxiety, and found that caregivers assumed a high level of pain before and during the procedure for their child, and the parent’s anxiety was significantly higher “before, during, and after” the procedure compared to the patient’s anxiety (aged 5 and older); this study only used ibuprofen as an analgesic [13]. Parent/guardian satisfaction influences overall experience since it is based more on the procedural care that the physician and ED team confers on the well-being of the patient. In this study, the participants stated that they would like to be involved in making medical decisions in the care of their children. Hence, a parent/guardian-centered approach should be combined with the ED team to complete the procedure. Such a collaboration would serve better to reduce anxiety, fear, and uncertainty for both the child and the caregiver.

Procedural sedation should be continuously monitored by maintaining continuous oxygen saturation, end-tidal CO2 detection, and the recording of vital signs, blood pressure, and heart rate. All these should also include other sedation assessment scales. Adverse effects of sedation like hypoventilation and hypoxia should be carefully monitored because although the medication dose is calculated based on patient weight and age there is a significant variation in response from one child to another. Reversal agents for anesthetics should also be available at the station/room where the procedure is being performed [14]. In a busy department, valuable human resources are monopolized by procedural sedation. The need for close monitoring and additional documentation may lead to increased hesitancy on the part of staff to perform sedation or the use of anxiolytics. Children who may fall asleep following the intranasal midazolam administration, for example, should be monitored with pulse oximetry at a minimum. The successful completion of the procedure utilizing these methods of sedation is sure to improve satisfaction both among the physicians and the parents [15].

This study faces several limitations. A relatively small sample size was surveyed at a single center. It was an ethnic parent/guardian population whose ages ranged between 22 and 40 years, which may not present a generalizable overview of anxiolytic use and its impact on EDs around the country. The education level of the majority of the participants was mainly in the high school and lower range. Such an age range may represent a limitation outside a metropolitan region. Some of the questions were phrased in a hypothetical fashion, where the participants were asked to assume the children received anxiolytic medication, and to answer a before and after laceration repair question. Thus, some parents with no experience with the actual anxiolytic medication may not have meaningfully understood the possible consequences of such a treatment.

## Conclusions

A review of both parent/guardian and physician preference on the use of anxiolytic medication techniques suggests that when properly employed, these practices may lead to improved comfort for both the children and their parent/guardian and may lead to improved outcomes for any simple laceration repair. While the use of anxiolytic medications presents inherent risks, we suggest that comprehensive protocols should be developed to facilitate the completion of procedures with a team approach that centers around the parent/guardian to allow them to participate in the workflow. A systemic management approach with appropriate monitoring and patient recovery may allow for an improved cosmetic outcome as well. It may also aid in reducing the inherent stress experienced by both the pediatric patient and their parent/guardian.
